# A New Stored Energy Model Based on Plastic Work of Back Stress during Cyclic Loading in Polycrystalline Metal

**DOI:** 10.3390/ma15155267

**Published:** 2022-07-30

**Authors:** Haifeng Xu, Xiaopeng Li, Wei Li, Peng Jiang, Yuanbo Zhao, Yinghonglin Liu

**Affiliations:** College of Mechanical Science and Engineering, Northeast Petroleum University, Daqing 163318, China; nepuxhf@163.com (H.X.); lixiaopeng19990317@163.com (X.L.); jpnepu@163.com (P.J.); zyb201507@126.com (Y.Z.); honglin_7799@163.com (Y.L.)

**Keywords:** stored energy, back stress, residual stress, fatigue damage, Ti-6Al-4V

## Abstract

Two mesomechanics models were analyzed in an attempt to reveal the relationship between stored energy and back stress. It has been indicated that the portion of elastic stored energy due to residual microstresses (*E*_SR_) is closely related to intergranular back stress (*X*_inter_), and the stored energy of dislocations inside grains (*E*_SD_) can be estimated with the plastic work of intragranular back stress (*X*_intra_). Then, the evolution of back stress during cyclic loading was studied, and the plastic work of back stress (*W*_pB_) was calculated with the low cycle fatigue experimental data of Ti-6Al-4V. The result shows that *W*_pB_ is partially released at every reverse loading, sufficient to reproduce the evolution of stored energy correctly under cyclic loading. The study also reveals that partially released energy is related to the decrease of *X*_inter_ at the initial state of reversal loading resulting from the reduction of the plastic strain incompatibility between grains.

## 1. Introduction

When a metal deforms plastically, most of the mechanical energy *W*_p_ expended in the deformation process is converted into heat *Q*. The remainder is stored in the material by creating and rearranging crystal imperfections, especially dislocations, stacking faults, etc. [1,2,3]. This is known as the stored energy of cold work *E*_S_, related to the damage leading to the initiation of fatigue microcracks [4,5].

*W*_p_ = *Q* + *E*_s_ (1)

**Figure 1:**
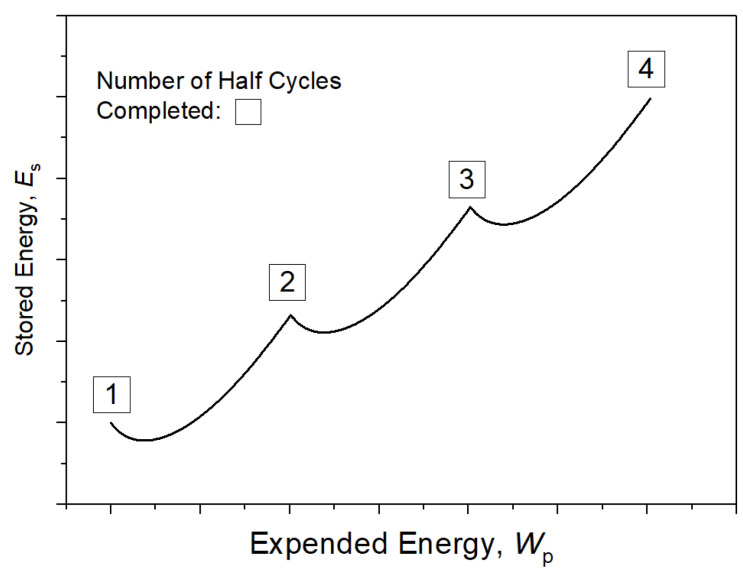
The evolution of stored energy versus accumulated plastic work during cyclic loading for metal.

The first attempts to describe stored energy were performed by Farren and Taylor [6]. Later, Taylor and Quinney [7] introduced the concept of stored energy and carried out a thorough study. An extensive review of the early work within this field was published by Bever et al. [8]. In these early studies, heat produced during deformation was measured using thermo-junctions or calorimeters. More attempts have been performed since then and today infrared radiometers or cameras are widely used [9,10,11,12]. Although there are differences in experimental methods, operators, and materials, the general trends are as follows:

1. The stored energy *E*_s_ is only a small part of the total plastic work *W*_p_: only 1% to 15% for the pure metals [8].
2. The ratio (*E*_s_/*W*_p_) is quite dependent on the strain level. In some cases [9,13], the ratio shows a maximum at minor plastic strains (less than 2%). Thereafter, the ratio decreases.
3. Under cyclic deformation, part of the stored energy is released at each reversed loading as shown in Figure 1 [14], the effect of which is believed to be closely related to the Bauschinger effect [8], or kinematic hardening [15].

**Figure 2:**
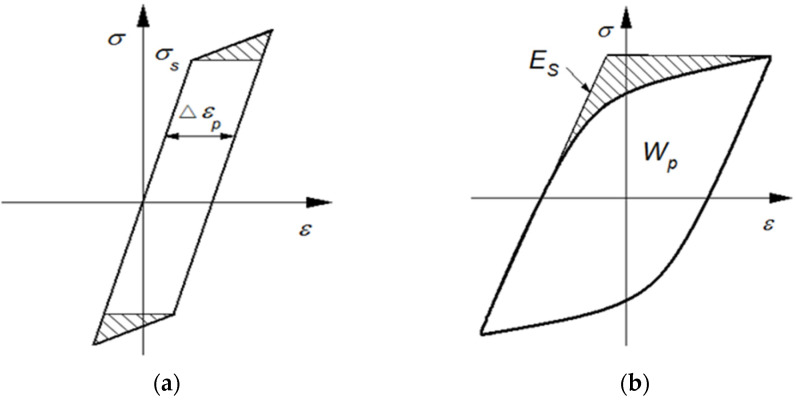
Stored energy models based on the stress–strain relation: (**a**) stored energy associated with working hardening; (**b**) stored energy connected with residual stress.

The experimental results give impetus to the theoretical description of energy balance in material. One of the possible ways to calculate stored energy is based on the stress–strain curve. Martin [16], who regarded the segment of the plastic work associated with work hardening as accumulating damage, first proposed a stored energy method from the stress–strain curve, as shown in Figure 2a with the shaded area. Later, a dislocation model, on the assumption of the irreversibility of dislocation motion, was established by Tanaka [17] to prove that the shaded area in Figure 2a is the stored energy related to the dislocations of pile-up in a single crystal. However, this method is not available for polycrystalline materials, especially for low cycle fatigue.

Another stored energy method from the stress–strain curve is proposed by Skelton [18,19,20] and Aravas [3]; they suggested that the shaded area in Figure 2b is the internal energy stored in the metal, which is famously known as “complementary plastic strain energy”. However, Szczepinski [21] and Oliferuk et al. [10] pointed out that this method can give us only part of the stored energy related to residual mircrostresses during non-homogeneous plastic deformation between grains (*E*_SR_), which is much lower than that corresponding to the creation of defects (mainly dislocations) inside grains (*E*_SD_). According to Szczepinski’s separation [21], the total stored energy is a sum of two parts:

*E*_S_ = *E*_SR_ + *E*_SD_ (2)

In order to determine the evolution of the stored energy, the evolution of the defect structure needs to be predicted for the given loading conditions. Chaboche [15] and Mollica et al. [22], Kamlah [23] and Cho et al. [24] calculated the stored energy by a continuum framework with internal variables used to characterize the defect evolution. They found that back stress is an essential internal variable in establishing their stored energy models.

With the fast development of computer technology, Benzerga et al. [2] developed a discrete dislocation plasticity model to calculate the stored energy of cold work associated with defects of dislocations. They also found that most of the energy stored in the dislocations is associated with their long-range stress fields (back stress), which accounted for more than 95%. Recently, mesoscopic microstructure-based modeling approaches such as crystal plasticity finite element method (CPFEM), have been popular for calculating the stored energy in a heterogeneous polycrystalline [25]. However, those methods are too complex to be used in engineering applications.

Both of the two classical stored energy methods shown in Figure 2 are related to flow stress, which is usually composed of friction stress *σ*_F_ and back stress *X*. The friction stress, corresponding to the stress required locally for a dislocation to move, is mainly related to the short-range obstacles [26]. Many scholars [27,28,29,30] believe that heat dissipation phenomena are related to dislocation movements in the material lattice (internal friction): overcoming short-range obstacles, while back stress refers to the stress associated with a local strain process providing long-range interactions with mobile dislocations. Thus, back stress is closely related to the stored energy, while the friction stress corresponds to the dissipated heat. In addition, back stress can be further divided into two components [31,32], namely intergranular back stress *X*_inter_, relating to the plastic strain incompatibilities between the different grains, and intragranular back stress *X*_intra_, linking to the dislocation distribution structures inside the grains. Thus,

*X* = *X*_inter_ + *X*_intra_ (3)

According to the definition, it is not difficult to discover that both *E*_SR_ and *X*_inter_ refer to the heterogeneous deformation between different microstructures, while both *E*_SD_ and *X*_intra_ correspond to the dislocation activities inside grains. Thus, this paper attempts to find the connection between back stress and energy storage by studying the role of back stress in constructing classical energy storage models.

## 2. Relationship between Stored Energy and Back Stress

### 2.1. Relationship between E_SR_ and X_inter_

**Figure 3:**
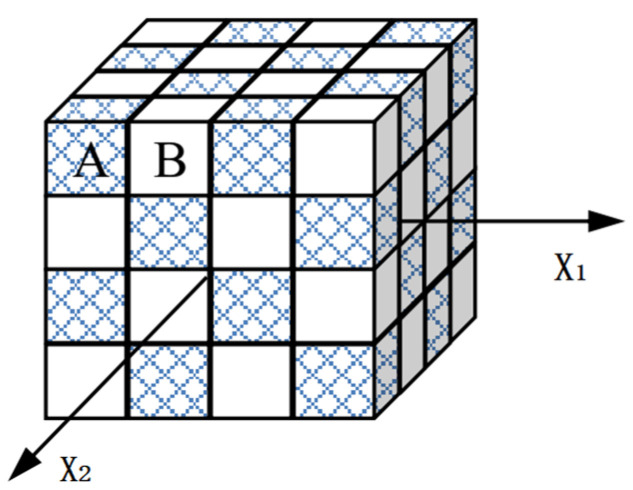
The mesomechanical model of polycrystalline material consisting two kinds of cuboidal elements with different yield stress.

**Figure 4:**
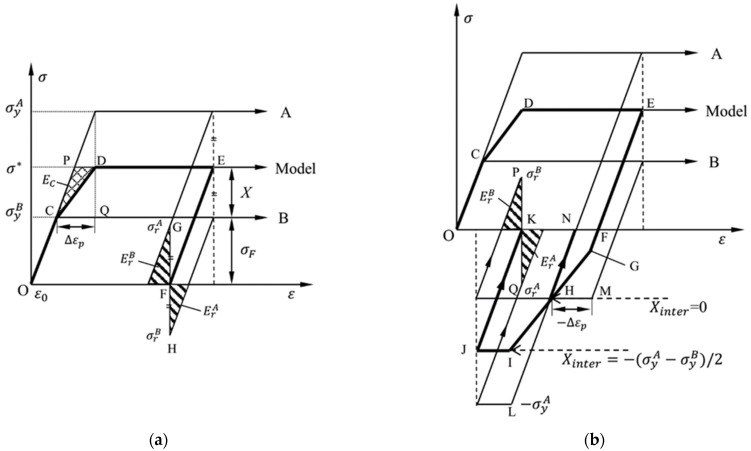
Stress–strain relation for the model is shown in Figure 3: (**a**) tension-going and (**b**) compression-going. The letters “A” and “B” represent two elements with different yield stresses $\sigma_{y}^{A}$ and $\sigma_{y}^{B}$.

The stored energy due to the residual microstresses remaining in a metal (*E*_SR_) has been studied by Szczepinski [21], on the basis of a simple mezomechanical model. This model represents a regular array of cuboidal A and B elements (Figure 3), based on the assumption that the elements deformed plastically without strain hardening, but each of them has a different yield stresses, $\sigma_{y}^{A}$ and $\sigma_{y}^{B}$, respectively, which cause non-homogeneous plastic deformation of the composite during uniaxial tensile loading, as illustrated in Figure 4. At the point of D, when the A element begins to deform plastically, the plastic strain of B element has been ∆*ε*_p_ greater than that of A element, and the plastic strain difference (∆*ε*_p_) remains unchanged during the subsequent deformation. At the point of E, the friction stress of the composite is $\sigma_{F}=\sigma_{y}^{B}$, and the back stress is

(4) $$X=(\sigma_{y}^{A}-\sigma_{y}^{B})/2$$

Note that this model excludes strain hardening of the A and B elements. Hence, the back stress arising during plastic deformation could only be related to plastic strain incompatibilities between grains, namely intergranular back stress *X*_inter_. From Equation (4), the larger the difference between $\sigma_{y}^{A}$ and $\sigma_{y}^{B}$, the greater value of *X*_inter_, which indicates that *X*_inter_ is a macro character of the microscopically structural inhomogeneity.

After unloading, the residual tensile stresses $\sigma_{r}^{A}$ remain in the A element while the residual compressive stresses $\sigma_{r}^{B}$ remain in the B element, as shown in Figure 4, and they are of the same absolute value:

(5) $$\sigma_{r}^{A}=-\sigma_{r}^{B}=(\sigma_{y}^{A}-\sigma_{y}^{B})/2$$

Moreover, they are also equal to *X*_inter_ in Equation (4). Thus, *X*_inter_ can be used to evaluate the magnitude of residual microstresses. The elastic energy per unit volume stored in the model due to the residual stresses remained in elements is equal:

(6) $$E_{\mathrm{SR}}=\frac{1}{2E}{\left(X_{\mathrm{inter}}\right)}^{2}$$

where $\sigma_{r}^{A}=-\sigma_{r}^{B}=X_{\mathrm{inter}}$ and *E* is the elastic modulus of elements. This energy density is equal to the complementary plastic strain energy density, *E*_C_, the shaded triangle ∆CDP in Figure 4, which can be easily estimated from the stress–strain diagram by measuring the area dashed in Figure 2b.

When the uniaxial loading direction is reversed, as shown in Figure 4b, the weaker B element first reaches its compressive yield stress $-\sigma_{y}^{B}$ and deforms plastically along M→H. In contrast, the A element remains in the elastic state and deforms along N→H, reducing the plastic strain difference (∆ε_p_) and releasing residual microstresses remaining in elements. At the point of H, where the two reversal loading curves intersect, the plastic strain difference reduces to zero, and the intergranular back stress *X*_inter_ = 0, as shown in Figure 4b. After unloading, there are no residual microstresses remaining in A and B elements anymore; That is, $\sigma_{r}^{A}=\sigma_{r}^{B}=X_{\mathrm{inter}}=0$, and no elastic energy is stored in the composite as well: $E_{\mathrm{SR}}=\frac{1}{2E}{\left(X_{\mathrm{inter}}\right)}^{2}=0$.

Thus, at the initial stage of reversal loading, the release of stored energy connected to the residual microstresses is attributed to decreased plastic strain incompatibility from grain to grain.

As the reversal loading continues, the stored energy increases again, as shown in Figure 4b. From H to I, the B element deforms plastically in the reversal direction. In contrast, the A element remains in the elastic state and deforms along H→L. Thus, the degree of the plastic strain incompatibility increases again between elements, which increases the absolute value of intergranular back stress |*X*_inter_|. At the point of J, the intergranular back stress

(7) $$X_{\mathrm{inter}}=-(\sigma_{y}^{A}-\sigma_{y}^{B})/2$$

as shown in Figure 4b. After unloading, residual compressive stresses $\sigma_{r}^{A}$ and residual tensile stresses $\sigma_{y}^{B}$ remain in the A and B elements, respectively:

(8) $$\sigma_{r}^{A}=-\sigma_{r}^{B}=X_{\mathrm{inter}}=-(\sigma_{y}^{A}-\sigma_{y}^{B})/2$$

Thus, during the subsequent reversal loading, the elastic energy per unit volume stored in the model due to residual stresses remaining in elements is $E_{\mathrm{SR}}=\frac{1}{2E}{\left(X_{\mathrm{inter}}\right)}^{2}$ equal to the stored energy during the tensile process.

Overall, in the mesomechanics model, our study on the process of loading and reversal loading reveals that the accumulation or dissipation of *E_SR_* is related to the increase or decrease of residual microstresses remaining in elements resulting from the increase or decrease of the degree of the plastic strain incompatibility between grains. While the |*X*_inter_| is a macroscopic representation of the residual microstresses, the *E*_SR_ can be expressed by *X*_inter_ in the formula form of Equation (6).

### 2.2. Relationship between E_SD_ and X_intra_

**Figure 5:**
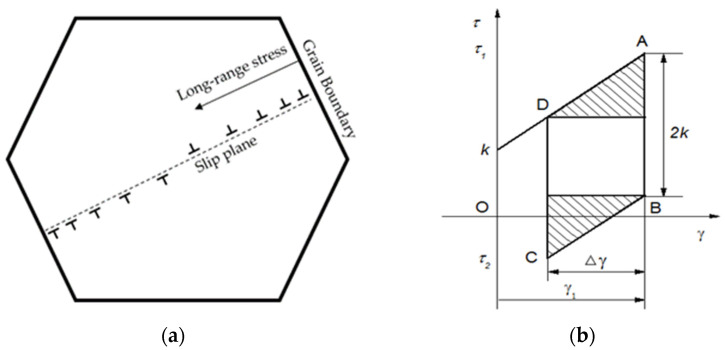
Stored energy model based on dislocation theory in a single crystal: (**a**) dislocation motion in a most favorably oriented grain; (**b**) stored energy in the stress–strain hysteresis loop. The *k* represents initial yield stress in a single crystal, while the Δ*γ* is the strain range.

In the real material, besides the energy stored due to residual microstresses (*E*_SR_), there is also the energy connected with the creation of defects (mainly dislocations) in particular grains (*E*_SD_). Thus, the strain-hardening effect inside grains should be accounted for. A dislocation model was introduced by Tanaka [17] to study the stored energy of dislocations within long-range stress fields inside a single crystal, as shown in Figure 5.

In this model, the dislocations generated in a most favorably oriented grain under the tensile stress are piled up against the grain boundary, as illustrated in Figure 5a, causing the long-range stress inside the grain, namely the dislocation stress *τ*^D^, corresponding to the intragranular back stress *X*_intra_. The dislocation stress *τ*^D^ and the plastic displacement *Φ*(*x*) depend on the dislocation density *D*(*x*). The stored energy of dislocations in unit volume has been given by Tanaka [17].

(9) $$E_{\mathrm{SD}}=U=-\frac{1}{2}\int_{-a}^{a}\tau^{D}\phi (x)dx$$

Using the inversion formula of Muskhelishvili, Equation (9) can be simplified as:

(10) $$E_{\mathrm{SD}}=\frac{1}{2}(\tau -k)\gamma =\frac{1}{2}\tau^{D}\gamma =\frac{1}{2}X_{\mathrm{intra}}\gamma$$

where *τ* is the loading of stress, *k* is the friction stress and *γ* is the plastic strain. Therefore, the amount of stored energy of dislocations (*E*_SD_) for a single crystal is equal to plastic work done by *X*_intra_, corresponding to the segment of the plastic work associated with work hardening as shown in Figure 5b by the shaded triangles.

The above analysis demonstrates that both *E_SR_* and *E_SD_* can be simply expressed by *X*_inter_ and *X*_intra,_ respectively, in Equations (6) and (10). Thus, it is reasonable to assume that there is a close connection between stored energy *E*_S_ and plastic work of back stress *W*_pB_ during working hardening.

## 3. Plastic Work of Back Stress during Cyclic Loading

During cyclic tests, the plastic work of back stress (*W*_pB_) is dependent on the evolution of back stress.

### 3.1. Hysteresis Loop of Back Stress

**Figure 6:**
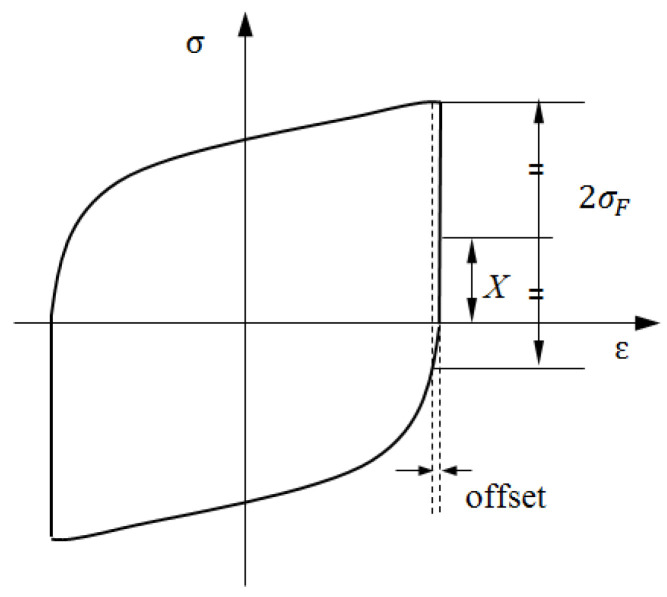
Determination of the back stress and the friction stress from the hysteresis loop.

From a mechanical point of view, the back stress, *X*, corresponds to the translation of the elastic domain, whereas the friction stress, *σ*_F_, represents the radius of this domain. Thus, according to Kuhlmann’s method [33], the back stress and friction stress can be extracted directly from the hysteresis loops, as schematically in Figure 6, where *σ*_F_ is determined at a reversed plastic strain offset (*ε*_offset_) within the range of 5 × 10^−6^~10^−3^, as suggested in references [32,34,35]. The back stress *X* can be thus obtained by *σ*_a_ = *X* + *σ*_F_. However, this method can only give out the internal stresses at the peak point.

**Figure 7:**
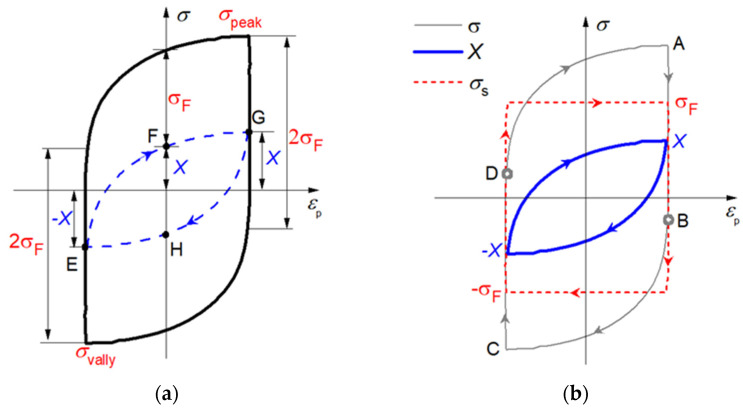
Internal stresses change around the loop. (**a**) back stress (blue dot lines); (**b**) friction stress (red dot lines).

During cyclic loading, Kuhlmann [33] also pointed out that the friction stress is approximately the same at the beginning and end of each half cycle with only slow changes from one cycle to the next, which indicate that only kinematic hardening (represented by *X*) is taken into account in a complete cycle. Thus, it is justifiable to assume that the size of the elastic domain remains unchanged and only the center of the yield surface travels along the loading path. In the case of kinematic hardening, the stress, at any given moment *t*, can be given by [36].

(11) $$\sigma =X+v\sigma_{F}$$

where v=±1, according to the direction of plastic strain, as illustrated in Figure 7a at A’ point. The circles in Figure 7a represent the yield surface with a fixed radius of *σ*_F_.

In the case of tension-going, $X=\sigma -\sigma_{F}$, the back stress increases from E to G along an upward locus $\hat{\mathrm{EFG}}$. While the loading direction reverses, $X=\sigma +\sigma_{F}$, the back stress decreases from G to E along a downward locus $\hat{\mathrm{GHE}}$ as shown in Figure 7a with dot lines. Let the short-range interaction stress (*σ*_S_) equal to the second term of the right-hand side of Equation (11): $\sigma_{S}=v\sigma_{F}$, then Equation (11) can be rewritten as

(12) $$\sigma =X+\sigma_{S}$$

The hysteresis loop of *σ*_S_ turns into a rectangle, as shown in Figure 7b with dot lines, and the hysteresis loop of *X* turns into a fusiform loop, as shown in Figure 7b with bold lines.

In Figure 7b, the total plastic work expended around the hysteresis loop is defined as ‘plastic strain hysteresis energy’, *W*_p_, and is given by $W_{p}=\int \sigma d\varepsilon_{p}$. According to Equation (12), the plastic work done by external stress can be divided into two components as follows:

(13) $$W_{p}=\int (\sigma_{S}+X)d\varepsilon_{p}=\int \sigma_{S}d\varepsilon_{p}+\int Xd\varepsilon_{p}$$

In the first item of Equation (13), the absolute value of *σ*_S_ is equal to the friction stress *σ*_F_ during cyclic plastic deformation; thus, it is defined as plastic work of friction stress, *W*_pF_.

(14) $$W_{\mathrm{pF}}=\int \sigma_{S}d\varepsilon_{p}$$

This work, corresponding to the shaped rectangle area of the loop in Figure 7b with dot lines, is related to the dissipated work against the friction stress resulting from the movement of dislocations: overcoming short-range obstacles [29].

The second item of Equation (13) is the plastic work done by the back stress, *W*_pB_.

(15) $$W_{\mathrm{pB}}=\int Xd\varepsilon_{p}$$

This work, corresponding to the shaped spindle area of the loop in Figure 7b, is associated with internal energy stored during the work-hardening process.

### 3.2. Accumulation and Dissipation of the Plastic Work of Back Stress

**Figure 8:**
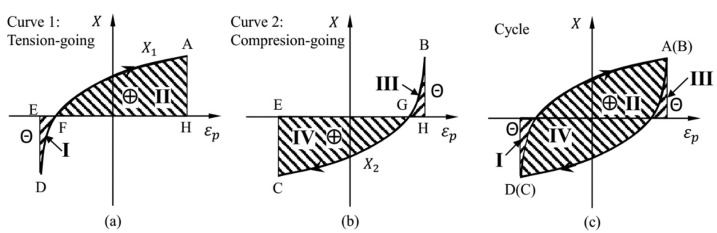
The accumulation and dissipation of plastic work of back stress during cyclic loading. (**a**) Curve1: Tension-going; (**b**) Curve2: Compression-going; (**c**) Closed loop. The symbol ⊕ represents the positive area, while the symbol ㊀ represents the negative area.

In the case of tensile-going loading, as shown in Figure 8a, the plastic work done by the back stress *W*_pB1_ can be easily integrated from *X*_1_-*ε*_p_ curve, as given by a definite integral of $\int_{\varepsilon_{\mathrm{pE}}}^{\varepsilon_{\mathrm{pH}}}X_{1}d\varepsilon_{p}$ for curve 1 according to the Equation (15). Mathematically, the definite integral is defined as the signed area of the region in the *X*-*ε*_p_ plane that is bounded by the graph of curve 1 and the *ε*_p_-axis and two vertical boundary lines. Thus, the area I below the *ε*_p_-axis subtracts from the total, while the area II above the *ε*_p_-axis adds to the total. However, during the compression-going loading, as shown in Figure 8b, the orientation of the definite integral of $\int_{\varepsilon_{\mathrm{pH}}}^{\varepsilon_{\mathrm{pE}}}X_{2}d\varepsilon_{p}$ for curve 2 is reversed; thus, the sign of the integral should be switched. Then, the area III above the *ε*_p_-axis subtracts from the total, while the area IV below the *ε*_p_-axis adds to the total. The negative areas I and III are related to the release of the previously cumulated plastic work of back stress when the absolute value of back stress |*X*| decreases to zero at each reverse loading. Finally, the total plastic work of back stress per cycle, ∆*W*_pB_, is equal to the area of the closed loop in Figure 8c.

To simplify the calculation, the plastic work of back stress per cycle, ∆*W*_pB_, can also be obtained by subtracting the plastic work of friction stress per cycle, ∆*W*_pF_, from the total plastic work per cycle, ∆*W*_p_.

(16) $$\Delta W_{\mathrm{pB}}=\Delta W_{p}-\Delta W_{\mathrm{pF}}$$

in which,

(17) $$\Delta W_{p}=\oint \sigma d\varepsilon_{p}$$

Since the friction stress is assumed to be a constant during a single complete cycle, ∆*W*_pF_ can be simply expressed by

(18) $$\Delta W_{\mathrm{pF}}=2\sigma_{F}\Delta \varepsilon_{p}$$

where ∆*ε*_p_ is the plastic strain range.

In our previous work [37], the low cycle fatigue experiments of Ti-6Al-4V alloy have already been carried out with a total strain-controlled mode, with a ratio of -1, a constant total strain rate of 4 × 10^−3^ s^−1^ and a triangular waveform performed on a computer-controlled 250 kN MTS810 closed-loop servo-hydraulic test machine at room temperature. Strain control was achieved by extensometer (12 mm gage length) arm tips located on the gage length. The strain amplitude chosen for the present tests ranges from 0.7% to 2.0%. The tests were continued until fracture. The correlation between the low cycle fatigue behavior of Ti-6Al-4V alloy and its microstructure evolution has been discussed in our previous work [37]. This paper will focus on the evolution of the plastic work of back stress during cyclic loading with different strain amplitudes.

**Figure 9:**
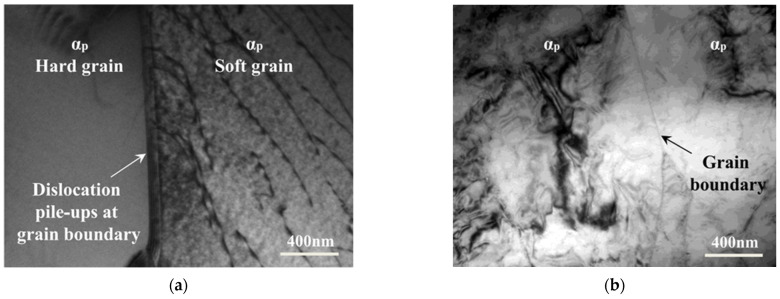
TEM observations of the fatigue specimen cycled to failure at (**a**) low strain amplitude of 0.7% and (**b**) high strain amplitude of 2.0%.

Transmission electron microscopy (TEM) examinations were also carried out to study the microstructure evolutions during cyclic deformations. At the small strain amplitude of 0.7%, the heterogeneous dislocation distribution between the adjoining α_p_ grains with different orientations is visible in Figure 9a. The grain boundary acts as a solid barrier to slips, so that the high density of dislocation lines observed in soft grain cannot cross through but only pile up at the grain boundary, which leads to a plastic strain incompatibility between microstructures and generates the long-range internal stresses between hard/soft grains, namely *X*_inter_. When the load is removed, the residual tensile stress remains in hard grains, and residual compressive stress remains in soft grains, as illustrated in Figure 4a. Associated with these residual microstresses is the elastic energy remaining in the internal structure after plastic deformation [21], i.e., *E*_SR_. The other part of stored energy, *E*_SD_, is related to the high density of dislocation lines in the soft grain and dislocation pile-ups at the grain boundary, which produces the internal stress inside soft α_p_ grain in Figure 9a, namely *X*_intra_. At the high strain amplitude of 2.0%, as shown in Figure 9b, homogeneous dislocation structure is observed in both of the two adjoining α_p_ grains, which indicates that plastic deformation of the Ti-alloy tends to homogenization from grain-to-grain throughout the microstructures. Furthermore, the density of dislocation inside α_p_ grains is larger than that at low strain amplitude. Thus, the contribution of residual microstresses will decrease with the increasing applied strain.

**Figure 10:**
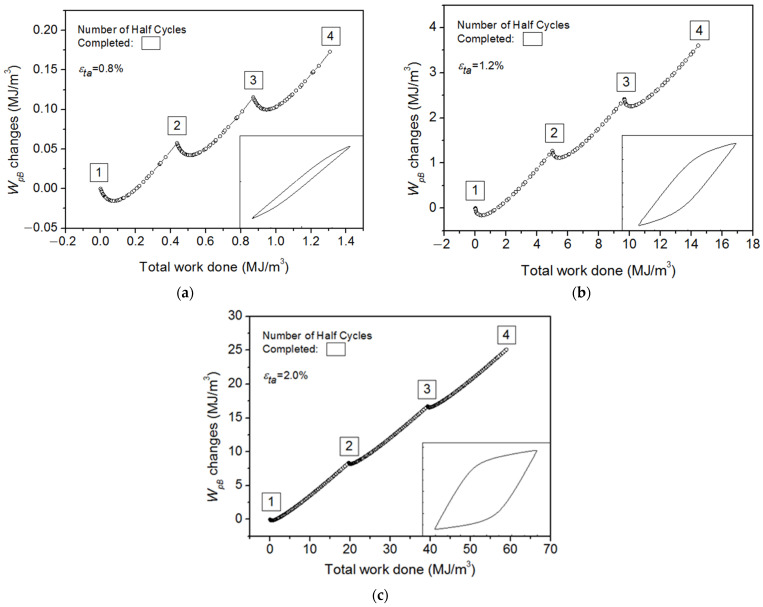
The accumulated plastic work of back stress vs. accumulated plastic strain energy for Ti-6Al-4V: (**a**) ε_ta_ = 0.8%; (**b**) ε_ta_ = 1.2%; and (**c**) ε_ta_ = 2.0%.

The back stress and friction stress have been extracted from the hysteresis loops with *ε*_offset_ = 10^−4^ in reference [37] according to Kuhlmann’s method [33]. The hysteresis loop of back stress can be easily calculated from Equation (12), in which *σ_S_* is a constant for each half cycle. Based on the numerical integration of *X*-*ε*_p_ cyclic loops, we can observe the evolution of *W*_pB_ and its partial released for each half cycle quantitatively. Figure 10a–c give out the evolution of *W*_pB_ as a function of the total plastic work *W*_p_ at strain amplitudes of 0.8%, 1.2%, and 2.0%, respectively. It is observed that the *W*_pB_ does not change monotonically during a half cycle, but instead decreases first and then increases in sequence. The dotted lines in Figure 10 predict very similar evolutions to the measurements made by Halford [14] as shown in Figure 1, i.e., that the stored energy is released at the very beginning of every reverse loading. According to the theoretical analysis in Section 2.1, the elastic energy connected with the residual microstresses (*E*_SR_) stored during the non-homogeneous plastic deformation is partially or completely released at every half cycle while |*X*_inter_| decreases to zero, resulting from the decrease of the degree of the plastic strain incompatibility from grain-to-grain at the initial stage of reversal deformation, which could also be used to explain the phenomenon of the release of stored energy during cyclic loading.

**Figure 11:**
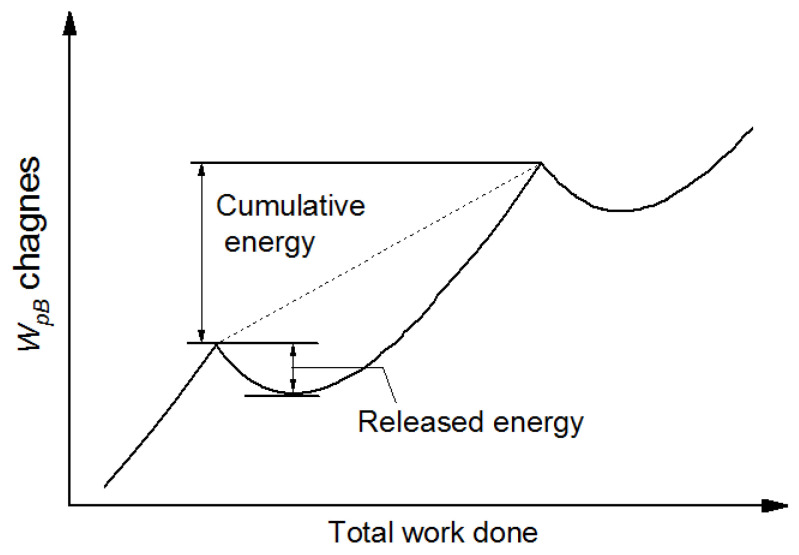
Schematic cumulative and released plastic work of back stress changes during one half cycle of completely reversed strain.

**Figure 12:**
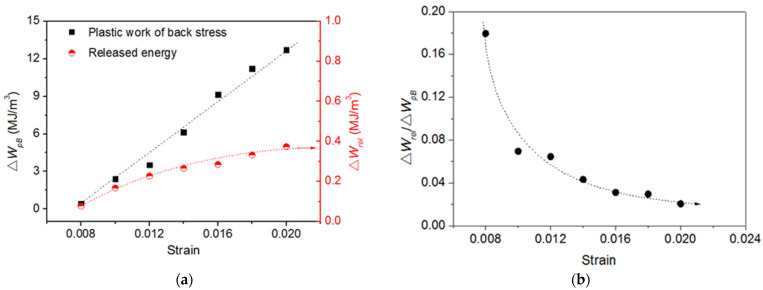
The relationship between ∆*W*_pB_ and ∆*W*_rel_: (**a**) the evolution of ∆*W*_pB_ and ∆*W*_rel_ at half-life versus strain; (**b**)the evolution of ∆*W*_rel_/∆*W*_pB_ versus strain.

It is convenient to separate the plastic work of back stress changes during a half cycle into a cumulative and a released component, as illustrated in Figure 11. By doing so, the released part of the plastic work of back stress (*W*_rel_), corresponding to the *E*_SR_, can be directly compared from test-to-test with different applied strains. Figure 12a shows the half-life plastic work of back stress per cycle ∆*W*_pB_ and its released energy per cycle ∆*W*_rel_ versus strain. The ∆*W*_pB_ increases linearly with increasing strain while ∆*W*_rel_ increases are gently first and then tends to be stable. Thus, as expected, the ratio of ∆*W*_rel_/∆*W*_pB_ drops exponentially from 18% to 2% for Ti-6Al-4V alloy with increasing strain (Figure 12b), which further confirmed that the proportion of *E*_SR_ decreases with increasing strain when the plastic deformation between heterogeneous microstructures tends to be more homogeneous.

**Figure 13:**
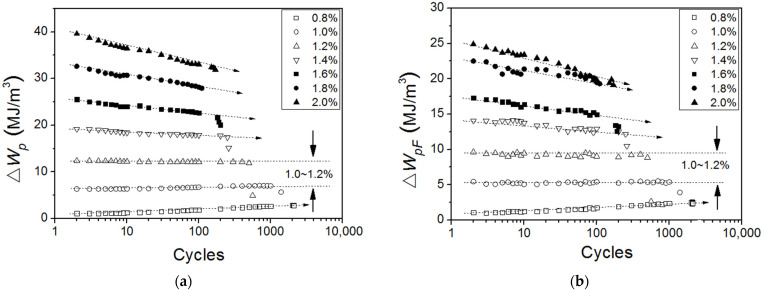

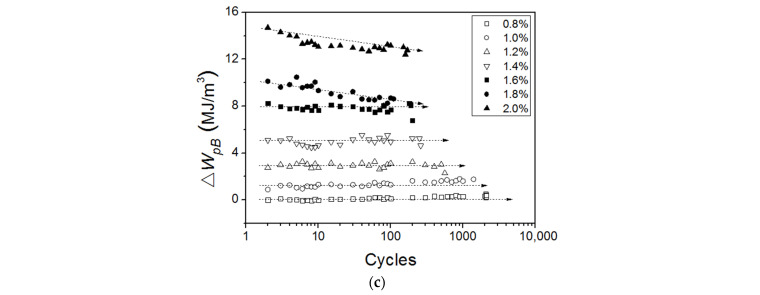
The evolution of (**a**) ∆*W*_p_, (**b**) ∆*W*_pF_ and (**c**) ∆*W*_pB_ with cyclic numbers.

**Figure 14:**
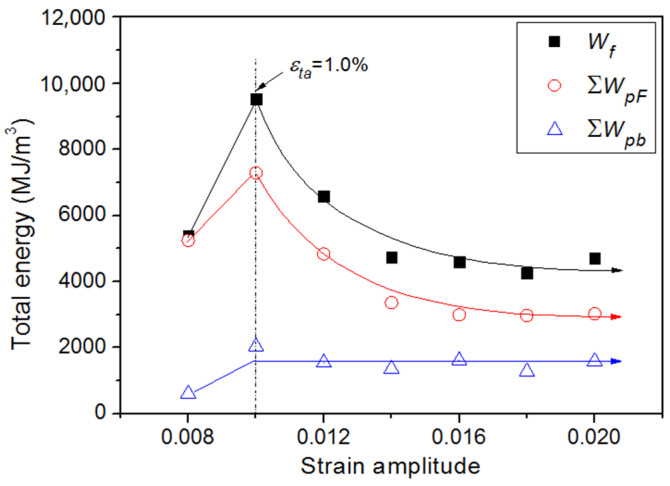
Total energy densities to failure.

According to the Equations (1), (3), and (4), the ∆*W*_p_, ∆*W*_pF_ and ∆*W*_pB_ have been calculated and displayed in Figure 13 as the function of the logarithm of the number of cycles at various applied strains. As can be noted from the comparison between Figure 13a,b, the evolution of ∆*W*_pF_ are consistent with that of ∆*W*_p_ at any applied strain. Both of them increase with cyclic numbers at small strain ranges (*ε*_ta_ ≤ 1.0~1.2%) and decrease at large strain ranges (*ε*_ta_ > 1.0~1.2%). However, the evolution of ∆*W*_pB_ has a different tendency, which stays almost constant with increasing cyclic numbers while *ε*_ta_ < 1.8%, and shows a decreasing tendency while *ε*_ta_ ≥ 1.8% (Figure 13c). The total hysteresis energy (or fatigue toughness) *W*_f_, the total plastic work of friction stress ∑*W*_pF_ and the total plastic work of back stress ∑*W*_pB_ up to failure for all specimens tested are also present in Figure 14. All of them show a maximum value at the strain amplitude of 1.0%, which corresponds to the breaking point of the bilinear behavior observed in the C-M plot of Ti-6Al-4V Alloy [37]. It may be seen from Figure 14 that both of *W*_f_ and ∑*W*_pF_ decrease exponentially with increasing applied strain at the strain range of *ε*_ta_ ≥ 1.0%, while ∑*W*_pB_ remains relatively constant over the strain range of *ε*_ta_ ≥ 1.0%. Wong et al. [38] also found that the total stored energy is almost constant while the total hysteresis energy varies over the fatigue life range. Thus, the plastic work of back stress is sufficient to correctly reproduce and explain the evolution of stored energy during cyclic loading.

## 4. Conclusions

In this work, two mesomechanics models were analyzed in an attempt to find the connection between back stress and stored energy. Then the evolution of back stress during cyclic loading was studied, and the plastic work of back stress (*W*_pB_) was calculated with the low cycle fatigue experimental data of Ti-6Al-4V. The main findings of this study can be summarized as follows:

(1) Based on the mesomechanics model, our study on the process of cyclic loading reveals that the partial release of stored energy is related to the decrease of residual microstresses remaining in elements resulting from the decrease of the degree of the plastic strain incompatibility between grains. Additionally, the |*X*_inter_| is a macroscopic representation of the residual microstresses.
(2) In Tanaka′s dislocation model, the amount of stored energy of dislocations (ESD) for a single crystal is equal to plastic work done by *X*_intra_.
(3) The plastic work of back stress (*W*_pB_) was calculated with the low cycle fatigue experimental data of Ti-6Al-4V. The results show that *W*_pB_ is partially released at every reverse loading, sufficient to reproduce the evolution of stored energy correctly under cyclic loading. Furthermore, the total plastic work of back stress ∑*W*_pB_ up to failure is almost constant over the strain range of *ε*_ta_ ≥ 1.0%, while the fatigue toughness *W*_f_ decrease exponentially with increasing applied strain. Thus, the plastic work of back stress is a more suitable damage criterion.

Is the plastic work of back stress the stored energy? Further theoretical analysis and experimental verification are still needed. The crystal plasticity finite element method (CPFEM) is a useful alternative for quantifying the stored deformation energy. It is also a great tool for investigating the relationship between plastic work of inter/intra back stress and inter/intra stored energy.

## Data Availability

Data can be provided upon request from the corresponding author.
